# HER-2/neu Marker Examination using Immunohistochemical Method in Patients Suffering from Gastric Adenocarcinoma

**Published:** 2013

**Authors:** Kourosh Movagharnejad, Majid Sharbatdaran, Shahryar Sheffaee, Mehrdad Kashifard, Sadegh Sedaghat

**Affiliations:** 1 *Department of Pathology, Rohani Hospital, Babol University of Medical Sciences, Babol, Iran.*; 2 *Cellular and Molecular Biology Research Center (CMBRC), Babol University of Medical Sciences, Babol, Iran.*; 3 *Department of Internal Medicine , Rohani Hospital, Babol University of Medical Sciences, Babol, Iran.*; 4 *Department of Hematology and Oncology, Rohani Hospital, Babol University of Medical Sciences, Babol, Iran.*

**Keywords:** HER-2/neu, gastric cancer, immunohistochemistry

## Abstract

Gastric adenocarcinoma is the second leading cause of death due to cancer in the world and in advanced stages the prognosis is poor even with current therapies. Over-expression of HER-2/neu has been seen in several cancers such as gastric cancer and its expression is associated with poor prognosis. The aim of this study is to evaluate the over-expression of HER-2/neu in gastric biopsy samples of patients with gastric carcinoma diagnosis; and to evaluate its probable relationship with clinical and pathological findings. The over-expression of HER-2/neu was examined retrospectively by immunohistochemistry method in 60 paraffin embedded samples collected in Babol, Iran, between 2010 and 2011. The over-expression of HER-2/neu has been observed in 6 patients (10%) and this over-expression was greater in the intestinal type of gastric adenocarcinoma than the diffuse type (12% vs. 6%); however, no statistically significant correlation between HER-2/neu expression and subtype, degree of differentiation, tumor type and age was observed. This over-expression was greater in differentiated types than undifferentiated types (18% vs. 5%).

Gastric adenocarcinoma is the most common gastric malignant tumor (1-2) and the most common cause of death due to cancer worldwide (3-5). Surgery is the most important treatment of this cancer, but it is efficient only in the early stages (6) and currently, prognosis for this cancer is still bad and 5 years survival period is between 5% and 25% (7-9). It was also shown that surgery, radiotherapy and chemotherapy had limited success in advanced grastic adenocarcinoma, indicating the necessity of other medical therapies in these cases (7, 10-12).

HER-2/neu protein is a transmembrane tyrosine kinase receptor, with 185 KDa weight belonging to the epidermal growth factor receptor family (EGFRs) (6, 13). This family consists of 4 members, including HER1, HER2, HER3 and HER4. These receptors have a common molecular structure that contains an extracellular ligand binding domain and an intracellular domain with tyrosine kinase activity (except for HER3) (2, 6).

In normal condition, ligand binding with specific growth factor leads to temporary activation of kinase and then, dimerization and tyrosine phosphorylation of several substrates, which are part of messaging cascade, will happen quickly. Oncogenic types of these receptors are capable of dimerization and innate activity without binding to growth factor; therefore, mutated receptors continuously transmit mutagenic messages into cells (2, 6).

In the last two decades, over-expression of HER-2/neu in different types of cancers has been seen, so that the over-expression of this marker was reported in about 10-34% of advanced breast cancer (14).

Also, in the different parts of the world, studies on over-expression of HER-2/neu in gastric adenocarcinoma show that it goes together with poor prognosis, increased invasion and metastasis. Likewise, this over-expression is more common in gastric adenocarcinoma than diffuse carcinoma subtype (15-16).

It also has been demonstrated that in gastric adenocarcinoma, expression of HER-2/neu is an independent prognostic factor for survival and prognosis (17-19).

Herceptin (Trustzumab) is a monoclonal antibody that specifically targets the HER-2/neu protein and connects to the extracellular receptor and it has been demonstrated that by its presence, treatment leads to improvement in prognosis and survival in patients suffering from gastric adenocarcinoma with over-expression of HER-2/neu (19-22).

The aim of this study was to examine the over-expression of HER-2/neu in patients suffering from gastric adenocarcinoma in Babol, Iran and to evaluate its probable correlation with clinico-pathological findings.

## Materials and Methods

**Patients**

Paraffin embedded blocks of 60 gastric biopsy samples with gastric adenocarcinoma diagnosis were collected during 2010-2011 in Pathology Departments of Rohani and Shahid Beheshti University Hospitals, Babol, Iran. Tissue sections were performed in order to perform H&E and immunohistochemical (IHC) staining. The subtype and grade of each tumor was identified based on H&E staining. The expression of HER-2/neu marker was assessed based on IHC staining.

**Immunohistochemistry**

**Fig. 1:**
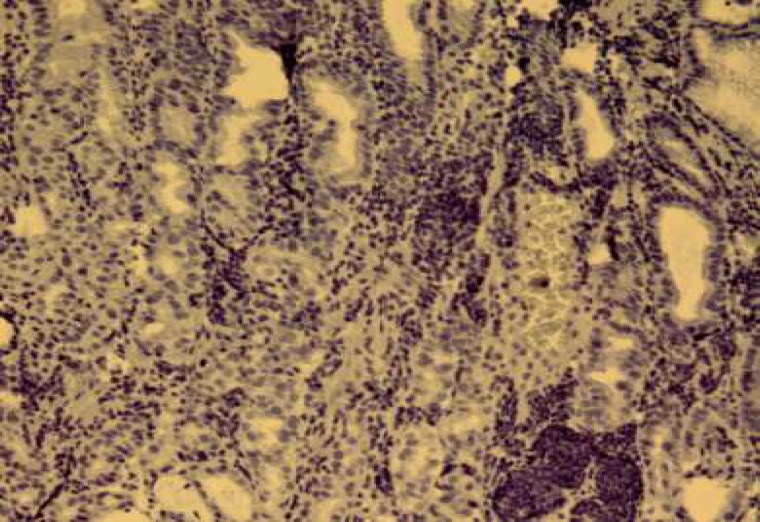
HER-2/neu negative

**Fig. 2:**
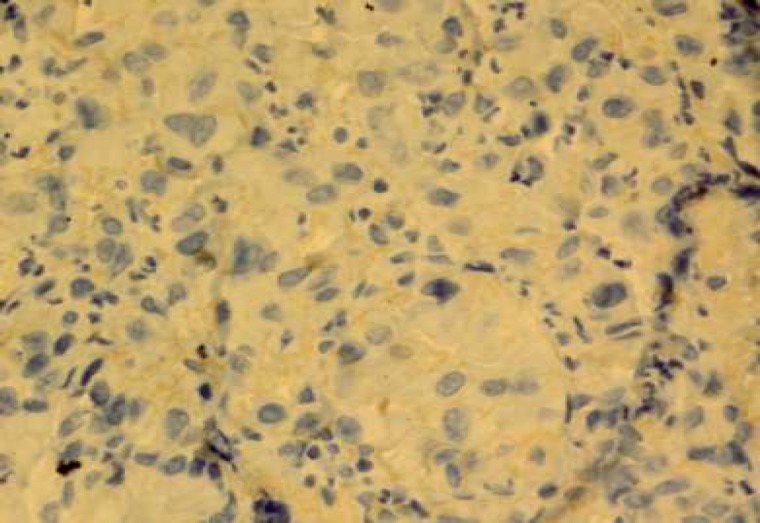
HER-2/neu overexpression score 1+.

**Fig. 3:**
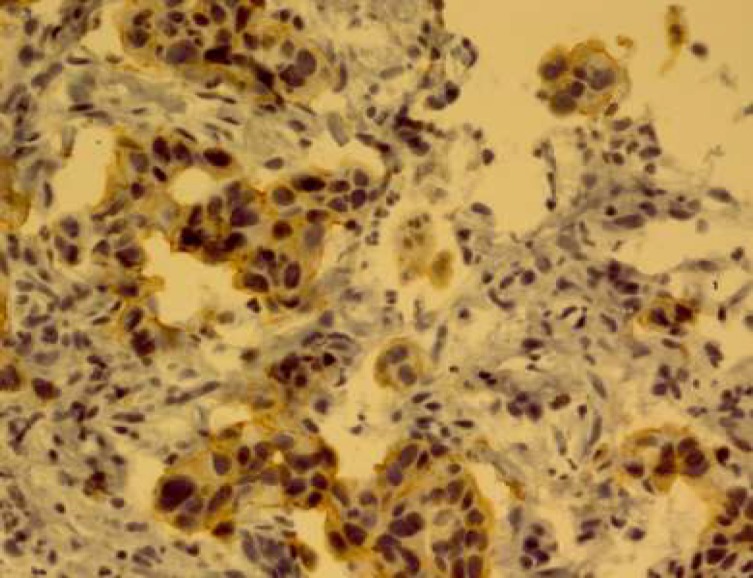
HER-2/neu overexpression score 2+.

**Fig. 4:**
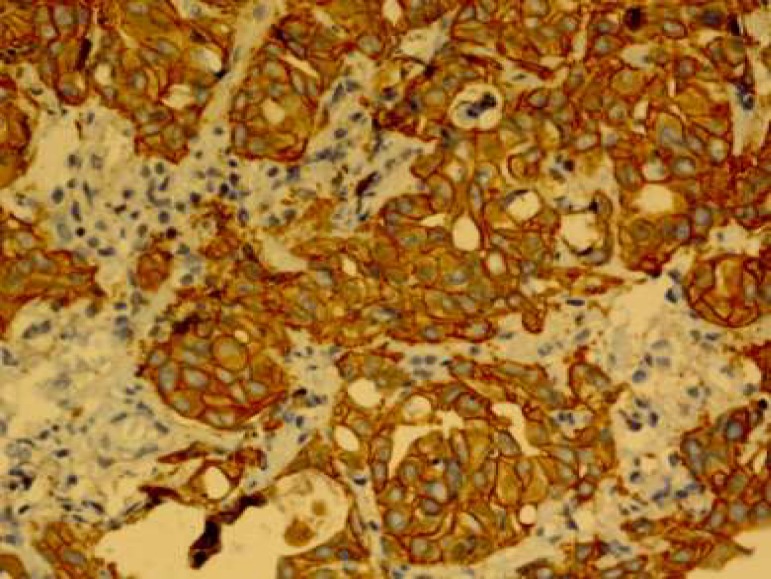
HER-2/neu overexpression score 3+.

The expression of HER-2/neu marker was examined by immunohistochemical staining by Envision method using Hercep Test Kit (Dako, Denmark) according to manufacture's instructions. Briefly, first, 3-4 microns sections of paraffin blocks were prepared and placed on sinalized slides. Then deparaffinisation and rehydration were performed and antigenic recovery was performed in a microwave oven in the presence of citrate buffer. After antigen retrieval, peroxidase was blocked to avoid endogenous peroxidase activity. Then staining with DAB chromogen followed by counterstaining with hematoxylin were performed (15). Also, a sample of breast carcinoma with over-expression of HER-2/neu was used (positive control) during coloration. Then, the slides were scored based on HercepTest Scoring in Dako instruction for gastric biopsies. Zero score was for the cases where the tumor cells or membrane were not stained. +1 score was for weak and diffuse membrane staining of tumor cell clusters (at least 5 cells). +2 score was for weak to moderate, lateral or basolateral complete membrane staining of tumor cell clusters; and+3 score was for strong, lateral or basolateral complete membrane staining of tumor cell clusters (Figures 1-4). 

Then cases with 0 and +1 scores were supposed as negative and+3 as positive (23). +2 cases which should be supposed as equivocal, due to impossibility of doing HER-2/FISH (Fluores-cence in situ Hybridization) were supposed as negative in this study.

**Statistical analyzes**

Data were analyzed using SPSS 17 software and X2 tests, Fisher more precise test and T-test. P< 0.05 was supposed to be statistically significant.

## Results

In this study, the age range of patients was between 35 to 94 years (average 70.9) and the male to female ratio was 2.57 (44 men and 16 women). 42 cases were intestinal subtypes (72%) and 18 cases were diffuse subtypes (30%). 28 cases (47%) were well-differentiated type, 12 cases (20%) were moderate differentiated and 20 cases (33%) were poorly differentiated. In 6 cases (10%), expression of HER-2/neu was positive. Clinico-pathological findings are shown in table 1.

## Discussion

Expression of HER-2/neu marker in gastric adenocarcinoma is different in various part of the world (15) and findings of the present study show that expression of HER-2/neu marker has been shown in 6 cases (10%) of 60 samples. The range of HER-2/neu expression in similar studies was reported between 5 to 62%. 

In the study of Femando et al. (2009) in Brazil, the HER-2/neu marker expression was 5% (2 cases of 37 samples) (15) and in another study conducted in Switzerland (2007) the expression of HER-2/neu marker was only 4.9% (24). In a study conducted in the USA (2008) by Gravalos et al., 22% of cases were HER-2/neu positive (6).

**Table 1:** Relationship between HER-2/neu expression and clinical and pathological findings in patients with gastric cancer

Pathological and clinical findings	number	Expression of HER-2/neu		P Value
		Positive	%	
male	44	5	%11	P> 0.05 Sex
Female	16	1	%6	
≥ 65 years	44	6	%14	P> 0.05 Age
> 65 years	16	0	%0	
Intestinal	42	5	%12	P> 0.05 Histological type
diffuse	18	1	% 6	
Well differentiated	28	5	%18	P> 0.05 Histological grade
Moderately differentiated	12	0	%0	
Poorly differentiated	20	1	%5	

The expression of HER-2/neu in intestinal type was more than diffuse type (12% vs. 6%). Despite these differences, there was no statistically significant difference (P> 0.05). This finding is in contrast with most studies conducted in other areas. For example, in a study conducted by Park et al. (2005), the expression of HER-2/neu marker in intestinal type was significantly (p<0.05) higher than the diffuse type (25). However, another research conducted by Jeung et al. (2012) did not find a significant difference (26). The differences observed between the current study and other reports may be due to the relatively low expression of HER-2/neu marker in this area and therefore, to achieve a significant difference, the sample size should be increased. Concerning the expression of HER-2/neu marker in various differentiated adenocarcinoma, despite a higher expression of this marker in well differentiated group, there was no statistically significant difference (P> 0.05) which was similar to some studies performed in other areas such as Korea (25). However, it is in contrast with some other studies such as that performed in Iran by Rezaei et al. (2007) in which a significant difference was observed (27).

The present study has also shown that the expression of HER-2/neu in men is more than women. However, the difference is not statistically significant (P> 0.05), but this study is in accordance with most previous reports (10, 28) although, more studies with larger sample sizes, may show a significant difference in this area.

The present study has shown that the expression of HER-2/neu in older patients (≥ 65 years) is more, but this difference is not statistically significant (P> 0.05). This is in accordance with a previous study performed in Iran (27), but then again, more studies in this area with larger sample sizes may show a significant difference.

We observed an over-expression of HER-2/neu in 10 % of patients with gastric adeno-carcinoma in Babol. Since +2 cases in IHC staining which should normally be evaluated by FISH method were considered as negative cases, it is likely that the expression of this marker is more than 10% and therefore, these people might receive monoclonal antibodies (Trastuzumab) therapy.

## Conflict of interest

Authors declared no conflict of interest.
